# Reduced Graphene Oxides Decorated NiSe Nanoparticles as High Performance Electrodes for Na/Li Storage

**DOI:** 10.3390/ma12223709

**Published:** 2019-11-10

**Authors:** Yan Liu, Xianshui Wang

**Affiliations:** Information and Engineering School, Wuhan University of Engineering Science, Wuhan 430200, China

**Keywords:** NiSe/rGO, hydrothermal method, anodes materials, sodium ion battery, lithium ion battery

## Abstract

A facile, one-pot hydrothermal method was used to synthesize Nickel selenide (NiSe) nanoparticles decorated with reduced graphene oxide nanosheets (rGO), denoted as NiSe/rGO. The NiSe/rGO exhibits good electrochemical performance when tested as anodes for Na-ion batteries (SIBs) and Li-ion batteries (LIBs). An initial reversible capacity of 423 mA h g^−1^ is achieved for SIBs with excellent cyclability (378 mA h g^−1^ for 50th cycle at 0.05 A g^−1^). As anode for LIBs, it delivers a remarkable reversible specific capacity of 1125 mA h g^−1^ at 0.05 A g^−1^. The enhanced electrochemical performance of NiSe/rGO nanocomposites can be ascribed to the synergic effects between NiSe nanoparticles and rGO, which provide high conductivity and large specific surface area, indicating NiSe/rGO as very promising Na/Li storage materials.

## 1. Introduction

Batteries are considered as very promising electrical energy storage technologies for integration of large-scale renewable energy. However, both the commercial Li-ion batteries (LIBs) and the newly developing Na-ion batteries (SIBs) cannot meet the metrics of electrical energy storage, in terms of lifetime, cost, and safety. In order to satisfy the demand, intensive research has been concentrated on developing new type electrode materials with better cyclability and higher capacity. [1,2,3,4,5,6,7] With respect to the anodes, graphite is a popular candidate for lithium storage anode. However, the low theoretical capacity limit the application to high energy density LIBs. What is more, graphite is not able to serve as sodium storage anode due to the mismatch of lattice parameters and sluggish kinetics of large Na^+^ in graphite host. [8,9] Therefore, developing high performance Na/Li storage anodes is of great significance.

Great efforts have been concentrated on seeking high theoretical capacity anode materials beyond graphite, including disordered carbon, [10,11,12] metal/alloy, [13,14,15,16] oxide [17,18,19], and sulfides. [20,21] Transition metal sulfides have been investigated as hopeful Na/Li Storage anode materials owing to their high theoretical capacity, such as FeS_2_, [22,23] MoS_2_, [24,25,26,27,28,29,30,31,32] Co_3_S_4_, [33,34] Ni_3_S_2_, [35,36,37,38] WS_2_ [39,40], and TiS_2_ [41,42] with the theoretical capacity of 895, 670, 704, 446.5, 432.3, and 958 mAh g^−1^, respectively. Transition metal selenides have very similar properties as transition metal sulfides, however there are seldom report about transition metal selenides as anode materials for LIBs and SIBs. For example, Zhang et al. prepared FeSe_2_ microspheres which delivered a stable capacity of 372 mA h g^−1^ after 2000 cycles at 1 A g^−1^ for sodium storage. [43] Yang et al. have reported porous hollow carbon spheres decorated with MoSe_2_ nanosheets, which have a capacity of about 684 mA h g^−1^ after 100 cycles at 1 A g^−1^ for lithium storage and a capacity of about 580 mA h g^−1^ after 100 cycles at 0.2 A g^−1^ for sodium storage. [44] Zhang et al. synthesized ZnSe-rGO nanocomposites by a simple hydrothermal process for lithium storage which exhibited a capacity of about 778 mA h g^−1^ after 400 cycles at 1 A g^−1^. [45] NiSe, a p-type semiconductor, has been widely used for photovoltaic devices. To the best of our knowledge, there is only Zhang et al. have reported core-shell NiSe/C composites used for LIBs and NIBs which delivers a capacity of about 280 mA h g^−1^/428 mA h g^−1^ after 50 cycles at 0.1 A g^−1^ for sodium/lithium storage. [46] Nevertheless, the reversible capacity and rate performance still need to improve. Yang et al. synthesized carbon-supported nickel selenide (Ni_0.85_Se/C) hollow nanowires by two-step hydrothermal process for sodium storage which exhibited a capacity of about 390 mA h g^−1^. However, the capacity still need to be upgraded considerably. Moreover, the complex synthesis route are not suitable for large-scale applications [47].

Like other transition metal selenides, NiSe suffers from its low electronic conductivity and the huge volume change during Na^+^/Li^+^ insertion/extraction process. So as to solve these problems, herein, the nanostructured NiSe anchored on the rGO nanosheets are synthesized by a simple hydrothermal approach. Owing to the synergic effects between NiSe nanoparticles and rGO, the NiSe/rGO nanocomposites exhibit a good sodium-storage and lithium-storage performance, offering a low cost and high performance anode material for Na/Li-ion batteries.

## 2. Experimental Section

### 2.1. Material Synthesis

GO was synthesized from natural graphite nanoflakes using a modified Hummer’s method. [48] NiSe/rGO was synthesized via a facile hydrothermal process. Typically, GO powder (72 mg) was dispersed in 36 mL distilled water using ultrasonication for 3 h. After ultrasonication, 290 mg Ni(NO_3_)_2_·6H_2_O and 110 mg SeO_2_ were added and mechanically stirred for 10 min, followed by the addition of 15 mL NH_3_·H_2_O and 30 mL N_2_H_4_·H_2_O. After more than 30 min mechanically stirring, the mixture precursor solution was transferred to a 100 mL Teflon-lined autoclave and heated at 200 °C for 10 h. The resultant slurry was centrifuged and washed several times with distilled water and absolute ethanol, respectively. Then, the products were collected after being dried in a vacuum oven at 80 °C overnight. For comparison, bare NiSe and rGO were synthesized via the same method, except without the addition of GO powders and Ni/Se sources, respectively.

### 2.2. Material Characterization

The samples were characterized by X-ray diffraction (XRD, PANalytical, Cu Kα Radiation), Scanning electron microscopy (SEM, FEI, Sirion 200, Hillsboro, OR, USA), Tansmission electron microscopy (TEM, FEI, Tecnai G2 F30S, Hillsboro, OR, USA), Raman spectra (Renishaw Invia, 514.5 nm, London, UK), Nitrogen adsorption–desorption isotherm (Micromeritics, TriStar II 3020, GA, USA), and X-ray photoelectron spectroscopy (XPS, Kratos, Axis Ultra DLD, Manchester, UK), thermogravimetric/differential scanning calorimetry (TG/DSC, Netzsch, STA 449 F5, Selb, Germany). The detailed material characterization is shown in Electronic Supplementary Information (ESI).

### 2.3. Electrochemical Measurements

Except for using the NaPF_6_/LiPF_6_ as Na/Li salt in electrolyte solution, the working electode was fabricated as same to the previous report. [49] Cyclic voltammogram (CV) measurements and electrochemical impedance spectra (EIS) were performed on a CHI 660E electrochemistry workstation. The galvanostatic charge/discharge measurements were carried out at room temperature with cutoff voltages of 0~3.0 V using a LAND battery test system. The detailed electrochemical measurements is exhibited in ESI. 

## 3. Results and Discussion

**Figure 1:**
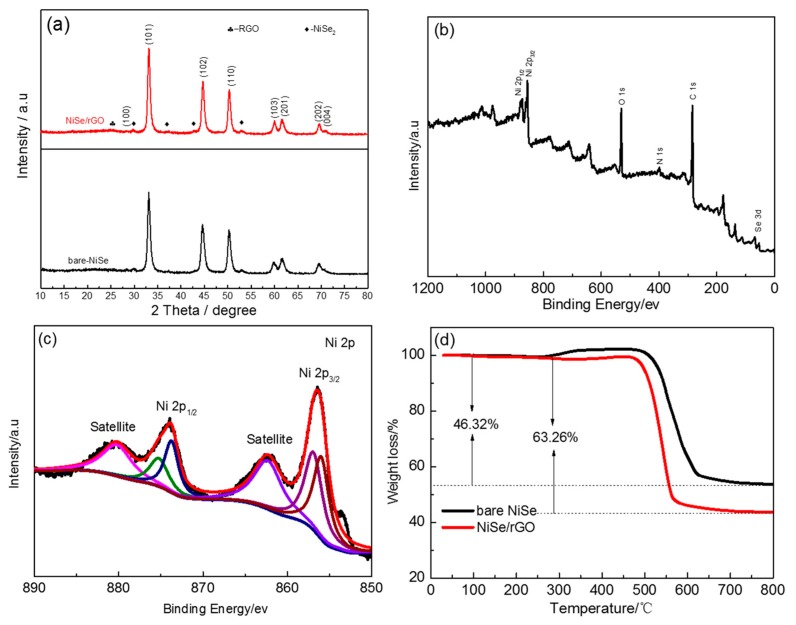
(**a**) X-ray diffraction (XRD) patterns of bare NiSe and NiSe/rGO composite. (**b**) X-ray photoelectron spectroscopy (XPS) survey and (**c**) high-resolution Ni 2p spectra of Nickel selenide (NiSe)/reduced graphene oxide nanosheets (rGO) composite. (**d**) thermogravimetric (TG) curves of the bare NiSe and NiSe/rGO.

The crystalline phases of NiSe/rGO and bare NiSe were identified by XRD, as shown in Figure 1a. The main phase of NiSe is indexed as a structure with hexagonal symmetry unit cell (JCPDS: 02-0892, P63/mmc space group, a = 0.366 nm, b = 0.366 nm and c = 0.533 nm). [46,50] The peaks at 29.8, 37.3, 42.9, and 53° in the XRD pattern can be assigned to NiSe_2_ (JCPDS: 41-1495). [51] Compared to that of bare NiSe, there is a broad peak observed at 20°~30° indicating the disordered stacking of rGO sheets, as shown in Figure S1. [30] according to the Debye Scherrer equation, the crystal size of NiSe/rGO and bare NiSe are calculated to be ~14 nm and 16 nm, respectively. A typical XPS survey pattern of NiSe/rGO is shown in Figure 1b and reveals the characteristic peaks of C, O, N, Ni, and Se. N is derived from N-dope of rGO with the source of N_2_H_4_·H_2_O. Figure 1c shows the high-resolution (HR) Ni 2p spectrum, which exhibit two intensive peaks at around 856.5 and 874 eV corresponding to the Ni 2p3/2 and Ni 2p1/2 levels, respectively. Meanwhile, there are two weak peaks at binding energies of 862.3 and 880.5 eV for Ni 2p3/2 satellites and Ni 2p1/2 satellites, respectively. [51] After refined fitting, the two peaks of Ni 2p at 856.0 and 874 eV are indexed to Ni^2+^, while the fitting peaks at 856.5 and 874.9 eV are indexed to Ni^4+^, which are in good agreement with the XRD result. The HR Se 3d spectrum (Figure S2a) exhibits two intensive peaks, one peak located at 54.8 eV attributed to Se^2-^ of NiSe and NiSe_2_, [52] and another peak located at 59.5 eV indexed to Se^4+^ of the impurity of SeO_2_. [53] As for the high-resolution C 1s spectrum (Figure S2b), the peaks at 283 eV, 286 eV, and 288.5 eV were assigned to C–C/C=C, C–O, and C=O bond, respectively. [51] The dominant C 1s peak at 283 eV indicates the reduction of GO during the hydrothermal process with N_2_H_4_·H_2_O.

Figure S3 presents the Raman spectra of GO and NiSe/rGO. The intensity ratio of D/G for the NiSe/rGO composite and GO were 0.95 and 1.26, respectively, also proves the reduction of GO to rGO with more defects and disordered structure. [51] Compared to rGO, the D and G bands of NiSe/rGO composite shift to lower wave number, which could be ascribed to the interaction between rGO and NiSe. Figure S4 shows the N_2_ adsorption-desorption isotherms of bare NiSe and NiSe/rGO. Both of bare NiSe and NiSe/rGO are identified as type IV isotherm (IUPAC), indicating the mesoporous structure. Base on the BET analysis, the specific surface areas of the bare NiSe and NiSe/rGO were calculated to be 4.6 and 15.6 m^2^ g^−1^, respectively. The large surface area of the NiSe/rGO not only increases the contact area of the electrode/electrolyte, but also facilitates the rapid insertion and diffusion of Na^+^/Li^+^. The content of rGO in NiSe/rGO is measured by TGA as shown in Figure 1d. For bare NiSe, 46.32% of weight is lost at about 520 °C due to the transformation of NiSe and NiSe_2_ to NiO in the air atmosphere. In contrast, NiSe/rGO exhibits a larger weight loss of 63.26%, which is equal to the total weight loss of NiSe and rGO. As depicted in the Equation below:

A × 63.26% = A × X × 46.32% + A × (1 − X)

A: The mass of NiSe/rGO. X: The pecentage of NiSe in NiSe/rGO composite. Therefore, the accurate loading of NiSe in the NiSe/rGO is calculated to be as high as 68.44%. 

**Figure 2:**
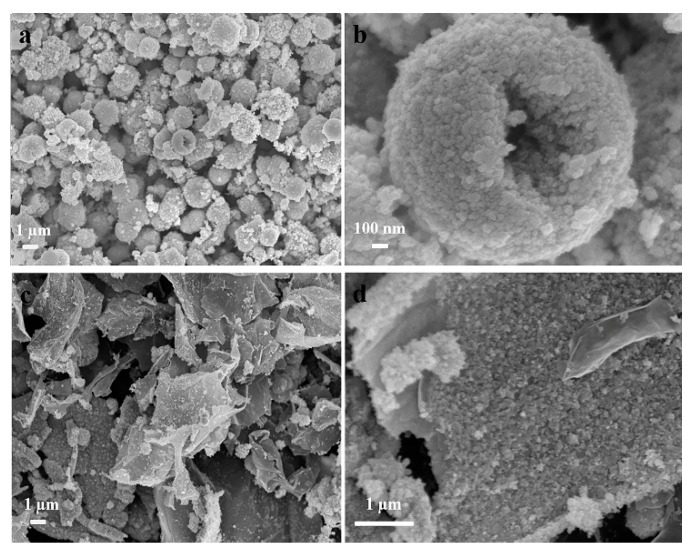
Field-emission scanning electron microscopy (FESEM) images of (**a**,**b**) bare NiSe and (**c**,**d**) NiSe/rGO composite.

The morphology of bare NiSe and NiSe/rGO are observed by field-emission scanning electron microscopy (FESEM), as shown in Figure 2. the bare NiSe mainly form hollow microspheres with diameters of 2–3 µm, which are assembled by NiSe nanoparticles with the sizes of about 20 nm (Figure 2a,b, Figure S5a). For NiSe/rGO composite, NiSe particles are distributed on the graphene nanosheets with some agglomeration consisting of some nanoparticles with sizes of about 17 nm (Figure 2c,d, Figure S5b).

**Figure 3:**
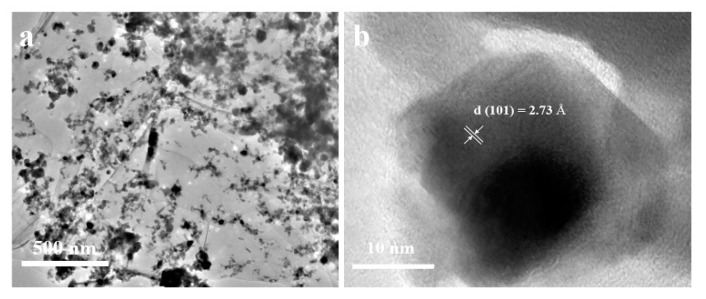
(**a**) TEM image of the NiSe/rGO; (**b**) high resolution TEM image of the NiSe/rGO.

Furthermore, the microstructure and particle size of the NiSe/rGO are analyzed by TEM and high resolution tansmission electron microscopy (HRTEM) (Figure 3, Figure S6). The low magnification TEM image (Figure 3a) presents a typical specimen of NiSe@graphene nanosheet. It can be seen that NiSe nanocrystals are anchored on the graphene nanosheets, which indicates that the rGO nanosheets greatly influence the morphology of NiSe. The SAED pattern (Figure S6, Table S1) presents the ring features, which can be well indexed as different crystal planes ((100), (101), (102), (103) (110), and (203)) of hexagonal structure, confirming the formation of well-crystallized NiSe. Figure 3b shows the HRTEM result of NiSe with lattice spacing of 0.287 nm agrees well with the (101) planes of NiSe and the crystal size of NiSe/rGO is about 20 nm which is basically consistent with XRD and SEM results.

**Figure 4:**
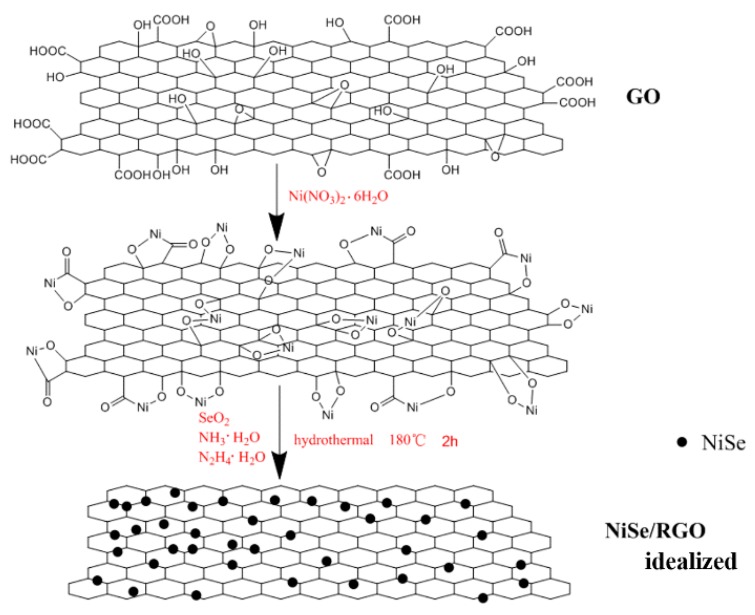
The illustration on growth processes of NiSe/rGO nanocomposites.

Based on the SEM, TEM analysis, and the previous literatures, [45,54] a likely synthesis mechanism of the NiSe/rGO nanocomposite is illustrated in Figure 4. Firstly, under vigorous ultrasonication in water, the precursor of GO is effectively exfoliated into GO sheets, and then Ni^2+^ ions are electrostatically combined with the negatively charged GO sheets through vigorous stirring in aqueous solution. The intermediate active species of Se^2−^ ions which are derived from SeO_2_ according to Equations (1) and (2) during the hydrothermal process and then react with Ni^2+^ ions to form NiSe (Equation (3)), meanwhile GO is reduced to rGO by N_2_H_4_·H_2_O. Obviously, graphene oxide nanosheets greatly influence the morphology of NiSe. In the absence of graphene, NiSe nanoparticles are assembled in the form of hollow nanospheres. With the graphene oxide, NiSe particles disperse on graphene nanosheets with some agglomeration. As a matter of fact, Figure 4 shows an ideal growth process. The NiSe nanoparticles exhibit some agglomeration rather than dispersed individual nanoparticles on the rGO sheets in our experiment.

(1) $${\mathrm{SeO}}_{2}+N_{2}H_{4}\cdot H_{2}O\to \mathrm{Se}+N_{2}+3H_{2}O$$

(2) $$3\mathrm{Se}+6{\mathrm{OH}}^{-}\to 2{\mathrm{Se}}^{2-}+{\mathrm{SeO}}_{3}^{2-}+3H_{2}O$$

(3) $${\mathrm{Ni}}^{2+}+{\mathrm{Se}}^{2-}\to \mathrm{NiSe}$$

**Figure 5:**
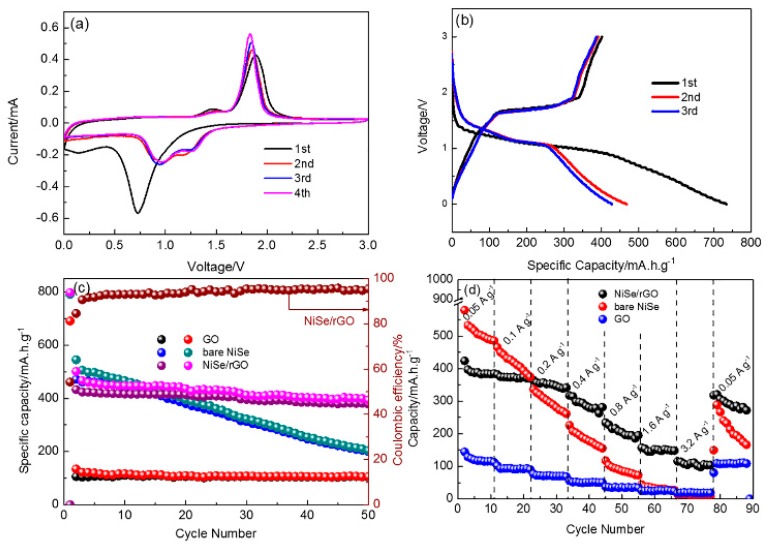
Sodium storage performances of as-prepared electrodes. (**a**) cyclic voltammograms of NiSe/rGO at a scan rate of 0.1 mV s^−1^; (**b**) the initial three cycles of NiSe/rGO composite electrode at 0.05 A g^−1^; (**c**) cycling performance; and (**d**) rate performance of NiSe/rGO composite, bare NiSe and rGO electrodes.

The Na-storage performance of as-prepared NiSe/rGO nanocomposites, bare NiSe and rGO are presented in Figure 5. The cyclic voltammetry measurements of NiSe/rGO are carried out in the range of 0~3 V as shown in Figure 5a. In the first cathodic scan, a major peak located at 0.75 V could be observed owing to the conversion reaction process (NiSe + 2Na^+^ + 2e^−^ → Ni + Na_2_Se) and formation of the solid electrolyte interphase (SEI) films. The reduction peak at 0.75 V disappears after the first cycle, and two new peaks located at around 1.0 V and 1.35 V appear. During the anodic scan, two peaks centered at 1.45 V and 1.80 V occur corresponding to the oxidation of metallic Ni to NiSe. [46] The overlapping of the CV curves since the second scan, indicates the high reversibility of NiSe/rGO composite electrode. Figure 5b shows the galvanostatic charge/discharge curves of NiSe/rGO anode at a current density of 0.05 A g^−1^ with voltage range of 0 and 3 V. The initial discharge curve displays an irreversible plateau at 0.75 V, in agreement with the CV features. In the following cycles, the charge/discharge profiles are nearly overlapping, implying that the good stability of NiSe/rGO electrode. Comparatively, the CV curve and galvanostatic charge/discharge curves of bare NiSe electrodes are very similar to NiSe/rGO, as shown in Figure S7, indicating composited with rGO do not change the electrochemical reaction process of NiSe. 

Figure 5c presents the cycling performance of as-prepared NiSe/rGO, bare NiSe and rGO electrodes at 0.05 A g^−1^. After two cycles activation, the NiSe/rGO composite electrode delivers a reversible capacity of 423 mAh g^−1^, which maintains at 378 mA h g^−1^ after 50 cycles with a capacity retention of 89.4% (compared with the second cycle), demonstrating the good cycling performance of NiSe/rGO. In comparison, bare NiSe and rGO electrodes exhibit much inferior performance. Bare NiSe has decent initial capacity but obvious fade with only capacity of 200 mA h g^−1^ after 50 cycles. The rGO electrode exhibits excellent cycling performance, but the capacity is only 105 mA h g^−1^.

The rate performances of NiSe/rGO, bare NiSe and rGO electrodes have been evaluated at various current densities ranging from 0.05 to 3.2 A g^−1^, which are presented in Figure 5d. At the current densities of 0.05, 0.1, 0.2, 0.4, 0.8, 1.6, and 3.2 A g^−1^, the NiSe/rGO composite electrodes deliver reversible capacities of 410, 375, 364, 314, 227, 152, and 110 mAh g^−1^, respectively. The reversible capacity can quickly recover to 320 mA h g^−1^ when the current density is returned to 0.05 A g^−1^ at. In comparison, bare NiSe and GO deliver capacities of 497, 394, 280, 177, 86, 32, and 15 mA h g^−1^ and 112, 92, 70, 51, 36, 24, and 19 mA h g^−1^ at the above-mentioned current densities, respectively. These results indicate much better high-rate performance is achieved by the compositing of NiSe with rGO.

**Figure 6:**
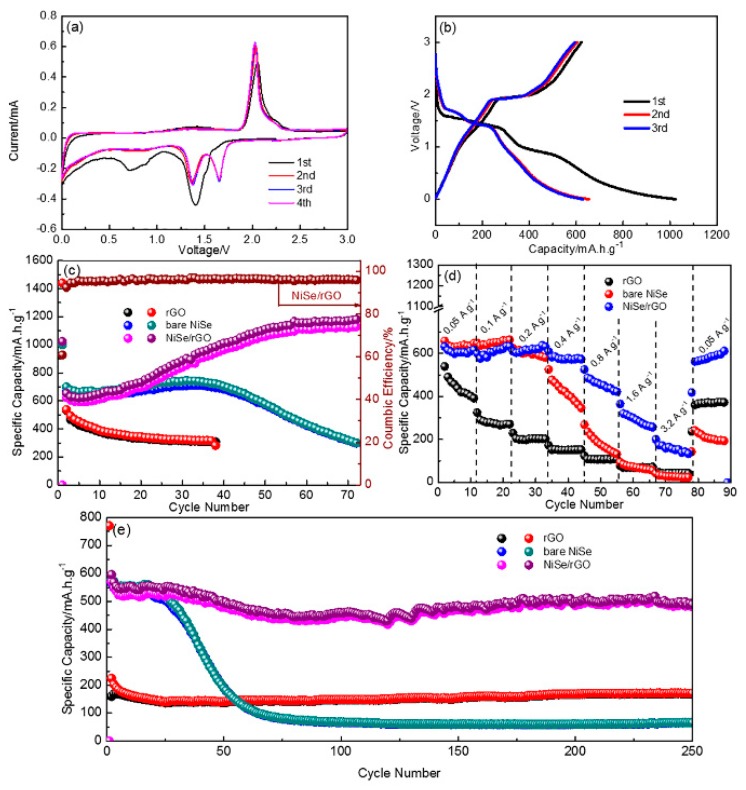
Electrochemical lithium storage performances of as-prepared electrodes. (**a**) cyclic voltammetry (CV) curves of NiSe/rGO at a scanning rate of 0.1 mV s^−1^; (**b**) the profiles of initial three cycles of NiSe/rGO at 0.05 A g^−1^; (**c**) cycling performance; (**d**) rate performance; and (**e**) long-term cycling performance of NiSe/rGO composite, bare NiSe, and rGO electrodes.

To extend the applications, the Li storage capability of as-prepared materials are also investigated with the coin-type half cells, as shown in Figure 6. Figure 6a exhibits the initial four cycles of CV for NiSe/rGO composite electrode, which are analogous to those of sodium storage. There are two cathodic peaks at around 1.3 and 1.7 V, and two anodic peaks at around 1.45 and 2.1 V. Figure 6b shows the galvanostatic charge/discharge curves of the NiSe/rGO at 0.05 A g^−1^. Compared with those of sodium storage, the voltage plateaus for lithium storage is relatively higher. In addition, the same to sodium storage, bare NiSe and NiSe/rGO electrodes have the similar CV curve and galvanostatic charge-discharge profiles as shown in Figure S8. Figure 6c presents the cyclability of different electrodes cycled at 0.05 A g^−1^. During the first cycle, the NiSe/rGO anode delivers 1023.2 and 622.8 mA h g^−1^ for discharge and charge processes with an initial coulombic efficiency of 61%. In the following cycles, there is a gradual increase of reversible capacity within the initial 50 cycles, indicating a long-lasting activation process. This phenomenon has been observed for transitional metal oxide electrodes in the literatures. [19,55,56] After 50 cycles, the NiSe/rGO anode exhibits very stable cycling performance with reversible capacity of 1125 mAh g^−1^. In comparison, the bare NiSe firstly exhibits a capacity of 668 mA h g^−1^. Although there is a slight increase of the capacity for NiSe electrode in the initial 30 cycles, the capacity quickly decays after 30 cycles, which is only around 290 mA h g^−1^ after 70 cycles. 

To test the rate performance, the NiSe/rGO composite, bare NiSe and rGO are tested at various current densities ranging from 0.05 to 3.2 A g^−1^ in 0~3.0 V as shown in Figure 6d. The NiSe/rGO anode demonstrates the best rate performance among all the tested electrodes, which delivers capacities of 573, 596, 602, 552, 448, 302, and 164 mA h g^−1^ at 0.05, 0.1, 0.2, 0.4, 0.8, 1.6, and 3.2 A g^−1^, respectively. The capacity can recover close to its initial capacity (589 mA h g^−1^) when the current densities switch from 3.2 to 0.05 A g^−1^. Furthermore, the discharge capacity of 250th cycle of NiSe/rGO at 0.4 A g^−1^ is 491mAh g^−1^, with the capacity retention of 86% compared with the second cycle, indicating the long-term cycling stability of NiSe/rGO electrode (Figure 6e). Comparatively, the bare NiSe anode delivers an initial capacity of about 566 mA h g^−1^, but quickly decays to 65 mA h g^−1^ after 250 cycles with capacity retention of only 11.5%. Although rGO exhibits very stable cyclability, it only delivers a capacity of only 171 mA h g^−1^.

**Figure 7:**
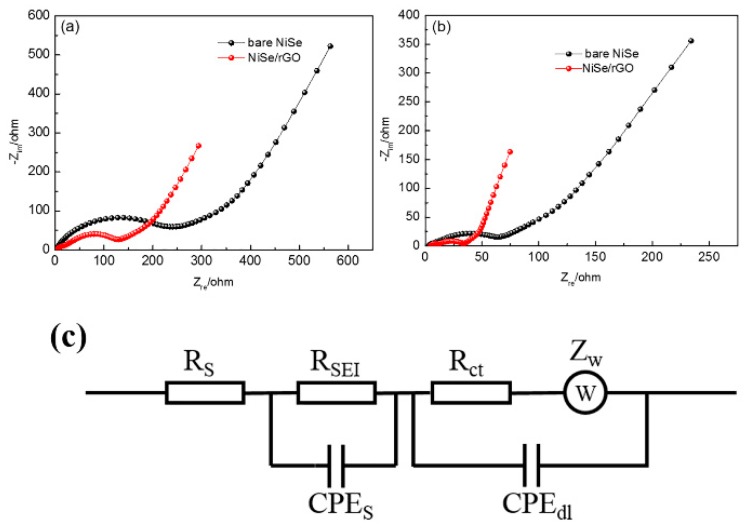
Electrochemical impedance spectroscopy (EIS) for the NIBs (**a**) and Li-ion batteries (LIBs) (**b**) with NiSe/rGO and bare NiSe electrodes after 10 cycles at a constant current density of 0.05 A g^−1^; (**c**) equivalent circuit model of the EIS spectra.

To understand why the NiSe/rGO anode exhibits superior electrochemical performances compared with the bare NiSe anode, electrochemical impedance measurements are carried out. A simulated equivalent electric circuit model is included. As shown in Figure 7 and Table 1, it can be easily found that NiSe/rGO exhibits smaller SEI surface resistance (R_SEI_) and charge-transfer resistance (R_ct_) than bare NiSe in both NIBs and LIBs, owing to the conducting frame of rGO in NiSe/rGO. The lower values of R_SEI_ and R_ct_ are considered to be key factors for improving the electrochemical performance of NiSe/rGO. It is noteworthy that the electrochemical impedance of bare NiSe and NiSe/rGO in LIBs are smaller than bare NiSe and NiSe/rGO in SIBs, indicating the easily inserted/extracted of lithium ion in the bare NiSe and NiSe/rGO electrodes. The fast diffusion kinetics is attributed to the small radius of lithium ions. The XRD pattern of NiSe/rGO electrode of SIB at fully-charged-state after 50 cycles, is shown in Figure S9. In this XRD pattern, the diffraction peaks of the NiSe/rGO electrodes can be well assigned to NiSe, NiSe_2_, rGO, and Cu (from the substrate of Cu foil), which are consistent to the NiSe/rGO composite before cycling (Figure 1a), indicating the NiSe/rGO electrodes with very good cycling stability. Moreover, SEM and TEM analysis are carried out to observe the microstructure changes of the bare-NiSe and NiSe/rGO electrodes (before and after 50 cycles) in SIBs. In Figure S10, the bare NiSe electrodes crash and agglomerate during the repeated cycling processes. In contrast, no significant morphology changes can be detected from the cycled NiSe/rGO electrodes, except for a little more agglomeration of NiSe particles compared with the pristine NiSe/rGO composite (Figure 3), further revealing the rGO can effectively buffer the volume change of NiSe nanoparticles.

Table S2 comparison of the electrochemical performance of NiSe/rGO with those of other anode materials in the literature. As shown in this table, the electrochemical performance of NiSe/rGO is superior to most of other anode materials. The good electrochemical performance of NiSe/rGO can be explained by the following three reasons: Firstly, the highly conductive rGO nanosheets would shorten the Na^+^/Li^+^ ions diffusion distance and improve the electron transport during the processes of charging and discharging. Secondly, being composited with rGO effectively enhances the specific surface area, which would offer more active sites for the accommodation of Na^+^/Li^+^. Finally, this composite structure of NiSe and rGO would effectively buffer the large volume changes during the sodium/lithium insertion/extraction processes.

## 4. Conclusions

In summary, NiSe/rGO nanocomposites are successfully synthesized by a one-step hydrothermal method and used for sodium and lithium storage. As a result, the as-prepared NiSe/rGO shows a high reversible capacity of 378 mA h g^−1^, and high rate capability of 110 mA h g^−1^ at 3.2 A g^−1^ for SIBs. For LIBs, it demonstrates a remarkable reversible capacity of 1125 mA h g^−1^ at 0.05 A g^−1^, good rate capability of 164 mA h g^−1^ at 3.2 A g^−1^, as well as superior capacity retention of 491 mA h g^−1^ at 0.4 A g^−1^ after 250 cycles. The enhanced electrochemical performance of the NiSe/rGO nanocomposites owes to the synergic effects between NiSe nanoparticles and rGO. The results indicate that the NiSe/rGO nanocomposites have great potential as anodes for sodium and lithium batteries.

## Figures and Tables

**Table 1:** Fitting results of the Nyquist plots using the equivalent circuit.

Samples	R_S_ (Ω)	R_SEI_ (Ω)	CPE_S_ (F)	R_ct_ (Ω)	CPE_dl_ (F)	Chi-Squared
Bare NiSe(Na)	3.0	25.3	5.4 × 10^−6^	171.7	1.3 × 10^−5^	3.0 × 10^−3^
NiSe/rGO(Na)	2.8	22.4	7.3 × 10^−6^	67.2	3.1 × 10^−5^	1.0 × 10^−3^
Bare NiSe(Li)	4.3	6.7	1.6 × 10^−6^	36.5	3.1 × 10^−5^	3.0 × 10^−3^
NiSe/rGO(Li)	2.1	4.1	3.9 × 10^−6^	18.2	8.5 × 10^−5^	1.9 × 10^−3^

## Supplementary Materials

The following are available online at https://www.mdpi.com/1996-1944/12/22/3709/s1.

## References

[1] Goodenough J.B., Park K.S. (2013). The Li-ion rechargeable battery: A perspective. J. Am. Chem. Soc..

[2] Goodenough J.B., Kim Y. (2010). Challenges for rechargeable Li batteries. Chem. Mater..

[3] Ellis B.L., Lee K.T., Nazar L.F. (2010). Positive electrode materials for Li-ion and Li-batteries. Chem. Mater..

[4] Bruce P.G., Scrosati B., Tarascon J.M. (2008). Nanomaterials for rechargeable lithium batteries. Angew. Chem. Int. Ed..

[5] Armand M., Tarascon J.-M. (2008). Building better batteries. Nature.

[6] Kang K., Meng Y.S., Bréger J., Grey C.P., Ceder G. (2006). Electrodes with high power and high capacity for rechargeable lithium batteries. Science.

[7] Tarascon J.-M., Armand M. (2001). Issues and challenges facing rechargeable lithium batteries. Nature.

[8] Scrosati B., Hassoun J., Sun Y.-K. (2001). Lithium-ion batteries. A look into the future. Energy Environ. Sci..

[9] Wu Y.P., Rahm E., Holze R. (2003). Carbon anode materials for lithium ion batteries. J. Power Sources.

[10] Liu H., Jia M., Wang M., Chen R., Sun N., Zhu Q., Wu F., Xu B. (2016). A floral variant of mesoporous carbon as an anode material for high performance sodium and lithium ion batteries. RSC Adv..

[11] Ma Q., Wang L., Xia W., Jia D., Zhao Z. (2016). Nitrogen-doped hollow amorphous carbon spheres@graphitic shells derived from pitch: New structure leads to robust lithium storage. Chem. Eur. J..

[12] Selvamani V., Ravikumar R., Suryanarayanan V., Velayutham D., Gopukumar S. (2016). Garlic peel derived high capacity hierarchical N-doped porous carbon anode for sodium/lithium ion cell. Electrochim. Acta.

[13] Zhou X., Yu L., Yu X.-Y., Lou X.W.D. (2016). Encapsulating Sn nanoparticles in amorphous carbon nanotubes for enhanced lithium storage properties. Adv. Energy Mater..

[14] Kim C., Lee K.-Y., Kim I., Park J., Cho G., Kim K.-W., Ahn J.-H., Ahn H.-J. (2016). Long-term cycling stability of porous Sn anode for sodium-ion batteries. J. Power Sources.

[15] Yi Z., Han Q., Zan P., Wu Y., Cheng Y., Wang L. (2016). Sb nanoparticles encapsulated into porous carbon matrixes for high-performance lithium-ion battery anodes. J. Power Sources.

[16] Wu L., Hu X., Qian J., Pei F., Wu F., Mao R., Ai X., Yang H., Cao Y. (2014). Sb–C nanofibers with long cycle life as an anode material for high-performance sodium-ion batteries. Energy Environ. Sci..

[17] Li W., Wang K., Cheng S., Jiang K. (2017). A two-dimensional hybrid of SbO_x_ nanoplates encapsulated by carbon flakes as a high performance sodium storage anode. J Mater. Chem. A.

[18] Li H.Z., Yang L.Y., Liu J., Li S.T., Fang L.B., Lu Y.K., Yang H.R., Liu S.L., Lei M. (2016). Improved electrochemical performance of yolk-shell structured SnO_2_@void@ C porous nanowires as anode for lithium and sodium batteries. J. Power Sources.

[19] Li D., Wang K., Tao H., Hu X., Cheng S., Jiang K. (2016). Facile synthesis of an Fe_3_O_4_/FeO/Fe/C composite as a high-performance anode for lithium-ion batteries. RSC Adv..

[20] Li H., Zhou M., Li W., Wang K., Cheng S., Jiang K. (2016). Layered SnS_2_ cross-linked by carbon nanotubes as a high performance anode for sodium ion batteries. RSC Adv..

[21] Yi Z., Han Q., Cheng Y., Wu Y., Wang L. (2016). Facile synthesis of symmetric bundle-like Sb_2_ S_3_ micron-structures and their application in lithium-ion battery anodes. Chem. Commun..

[22] Liu J., Wen Y., Wang Y., van Aken P.A., Maier J., Yu Y. (2014). Carbon-encapsulated pyrite as stable and earth-abundant high energy cathode material for rechargeable lithium batteries. Adv. Mater..

[23] Hu Z., Zhu Z., Cheng F., Zhang K., Wang J., Chen C., Chen J. (2015). Pyrite FeS_2_ for high-rate and long-life rechargeable sodium batteries. Energy Environ. Sci..

[24] Zhang L., Wu H.B., Yan Y., Wang X., Lou X.W. (2014). Hierarchical MoS_2_ microboxes constructed by nanosheets with enhanced electrochemical properties for lithium storage and water splitting. Energy Environ. Sci..

[25] Zhou X., Wan L.J., Guo Y.G. (2013). Synthesis of MoS_2_ nanosheet–graphene nanosheet hybrid materials for stable lithium storage. Chem. Commun..

[26] Xu X., Fan Z., Yu X., Ding S., Yu D., Lou X.W.D. (2014). Nanosheets-on-channel architecture constructed from MoS_2_ and CMK-3 for high-capacity and long-cycl-life lithium storage. Adv. Energy Mater..

[27] Zhu C., Mu X., van Aken P.A., Yu Y., Maier J. (2014). Single—Layered ultrasmall nanoplates of mos_2_ embedded in carbon nanofibers with excellent electrochemical performance for lithium and sodium storage. Angew. Chem. Int. Ed..

[28] Ding S., Zhang D., Chen J.S., Lou X.W. (2012). Facile synthesis of hierarchical MoS_2_ microspheres composed of few-layered nanosheets and their lithium storage properties. Nanoscale.

[29] Wang J., Liu J., Chao D., Yan J., Lin J., Shen Z.X. (2014). Self-assembly of honeycomb-like MoS_2_ nanoarchitectures anchored into graphene foam for enhanced lithium-ion storage. Adv. Mater..

[30] Xie X., Ao Z., Su D., Zhang J., Wang G. (2015). MoS_2_/Graphene composite anodes with enhanced performance for sodium-ion batteries: The role of the two-dimensional heterointerface. Adv. Funct. Mater..

[31] Choi S.H., Ko Y.N., Lee J.-K., Kang Y.C. (2015). 3D MoS_2_–graphene microspheres consisting of multiple nanospheres with superior sodium ion storage properties. Adv. Funct. Mater..

[32] Ryu W.H., Jung J.W., Park K., Kim S.J., Kim I.D. (2014). Vine-like MoS_2_ anode materials self-assembled from 1-D nanofibers for high capacity sodium rechargeable batteries. Nanoscale.

[33] Mahmood N., Zhang C., Jiang J., Liu F., Hou Y. (2013). Multifunctional Co_3_S_4_/graphene composites for lithium ion batteries and oxygen reduction reaction. Chem. Eur. J..

[34] Du Y., Zhu X., Zhou X., Hu L., Dai Z., Bao J. (2015). Co_3_S_4_ porous nanosheets embedded in graphene sheets as high-performance anode materials for lithium and sodium storage. J. Mater. Chem. A.

[35] Zhao Y., Feng J., Liu X., Wang F., Wang L., Shi C., Huang L., Feng X., Chen X., Xu L. (2014). Self-adaptive strain-relaxation optimization for high-energy lithium storage material through crumpling of graphene. Nat. Commun..

[36] Li D., Li X., Hou X., Sun X., Liu B., He D. (2014). Building a Ni_3_S_2_ nanotube array and investigating its application as an electrode for lithium ion batteries. Chem. Commun..

[37] Zhou W., Zheng J.-L., Yue Y.-H., Guo L. (2015). Highly stable rGO-wrapped Ni_3_S_2_ nanobowls: Structure fabrication and superior long-life electrochemical performance in LIBs. Nano Energy.

[38] Shang C., Dong S., Zhang S., Hu P., Zhang C., Cui G. (2015). A Ni_3_S_2_-PEDOT monolithic electrode for sodium batteries. Electrochem. Commun..

[39] Chen R., Zhao T., Wu W., Wu F., Li L., Qian J., Xu R., Wu H., Albishri H.M., Al-Bogami A.S. (2014). Free-standing hierarchically sandwich-type tungsten disulfide nanotubes/graphene anode for lithium-ion batteries. Nano Lett..

[40] Su D., Dou S., Wang G. (2014). WS_2_@ graphene nanocomposites as anode materials for Na-ion batteries with enhanced electrochemical performances. Chem. Commun..

[41] Trevey J.E., Stoldt C.R., Lee S.-H. (2011). High power nanocomposite TiS_2_ cathodes for all-solid-state lithium batteries. J. Electrochem. Soc..

[42] Ryu H.-S., Kim J.-S., Park J.-S., Park J.-W., Kim K.-W., Ahn J.-H., Nam T.-H., Wang G., Ahn H.-J. (2013). Electrochemical properties and discharge mechanism of Na/TiS_2_ cells with liquid electrolyte at room temperature. J. Electrochem. Soc..

[43] Zhang K., Hu Z., Liu X., Tao Z., Chen J. (2015). FeSe_2_ microspheres as a high-performance anode material for Na-ion batteries. Adv. Mater..

[44] Yang X., Zhang Z., Fu Y., Li Q. (2015). Porous hollow carbon spheres decorated with molybdenum diselenide nanosheets as anodes for highly reversible lithium and sodium storage. Nanoscale.

[45] Zhang Z., Fu Y., Yang X., Qu Y., Li Q. (2015). Nanostructured ZnSe anchored on graphene nanosheets with superior electrochemical properties for lithium ion batteries. Electrochim. Acta.

[46] Zhang Z., Shi X., Yang X. (2016). Synthesis of core-shell NiSe/C nanospheres as anodes for lithium and sodium storage. Electrochim. Acta.

[47] Yang X., Zhang J., Rogach A.L. (2018). Carbon-supported nickel selenide hollow nanowires as advanced anode materials for sodium ion batteries. Small.

[48] Kovtyukhova N.I., Ollivier P.J., Martin B.R., Mallouk T.E., Chizhik S.A., Buzaneva E.V., Gorchinskiy A.D. (1999). Layer-by-layer assembly of ultrathin composite films from micron-sized graphite oxide sheets and polycations. Chem. Mater..

[49] Tao H., Zhou M., Wang K., Cheng S., Jiang K. (2017). Nickel sulfide nanoparticles anchored on reduced graphene oxide in situ doped with sulfur as a high performance anode for sodium-ion battery. J. Mater. Chem. A.

[50] Xu K., Ding H., Jia K., Lu X., Chen P., Zhou T., Cheng H., Liu S., Wu C., Xie Y. (2016). Solution-liquid-solid synthesis of hexagonal nickel selenide nanowire arrays with a nonmetal catalyst. Angew. Chem. Int. Ed..

[51] Cho J.S., Lee S.Y., Kang Y.C. (2016). First introduction of NiSe_2_ to anode material for sodium-ion batteries: A hybrid of graphene—wrapped NiSe_2_/C porous nanofiber. Sci. Rep..

[52] Mandale A., Badrinarayanan S., Date S., Sinha A. (1984). Photoelectron-spectroscopic study of nickel, manganese and cobalt selenides. J. Electron. Spectrosc..

[53] Malmsten G., Thorén I., Högberg S., Bergmark J., Karlsson S., Rebane E. (1971). Selenium compounds studied by means of ESCA. Phys. Scr..

[54] Zhou T., Pang W.K., Zhang C., Yang J., Chen Z., Liu H.K., Guo Z. (2014). Enhanced sodium-ion battery performance by structural phase transition from two-dimensional hexagonal-SnS_2_ to orthorhombic-SnS. ACS Nano.

[55] Hu J., Sun C.F., Gillette E., Gui Z., Wang Y., Lee S.B. (2016). Dual-template ordered mesoporous carbon/Fe_2_O_3_ nanowires as lithium-ion battery anodes. Nanoscale.

[56] Zhao X., Vail S.A., Lu Y., Song J., Pan W., Evans D.R., Lee J.J. (2016). Antimony/graphitic carbon composite anode for high-performance sodium-ion batteries. ACS Appl. Mater. Interfaces.

