# Supplementary material for: Reduced Graphene Oxides Decorated NiSe Nanoparticles as High Performance Electrodes for Na/Li Storage
Source: Materials (Basel). 2019 Nov 10;12(22):3709. doi: 10.3390/ma12223709 (PMC6888247; doi:10.3390/ma12223709)
Supplement: Supplementary file 1 [file materials-12-03709-s001.pdf]

materials

Supporting Information

Reduced Graphene Oxides Decorated NiSe Nanoparticles as High Performance electrodes for Na/Li Storage

Yan Liu and Xianshui Wang *

Information and Engineering School, Wuhan University of Engineering Science, Wuhan 430200, China; qiyan_liu@126.com
* Correspondence: xianshui_wang@126.com; Tel.: 027-8182-0304

1. The Detailed Material Characterization and Electrochemical Measurements Process

Material Characterization

The crystalline structure of as-synthesized materials was recorded by powder X-ray diffraction (XRD) using a PANalytical Multi-Purpose Diffractometer equipped with a Cu Kα Radiation (λ = 1.5406 Å). Particle morphologies were characterized by field-emission scanning electron microscopy (FESEM, FEI, Sirion 200, Hillsboro, OR, USA) coupled with an energy-dispersive X-ray (EDX, Oxford Instrument, Oxfordshire, UK) spectrometer. The morphology was further analyzed by transmission electron microscopy (TEM, FEI, Hillsboro, OR, USA) using a Tecnai G2 F30S-Twin microscope. Raman spectra were collected with a Renishaw Invia spectrometer (London, UK) using Ar^+ laser of 514.5 nm at room temperature. The specific surface areas were determined by Brunauer–Emmett–Teller (BET) nitrogen adsorption–desorption measurement on TriStar II 3020 (Micromeritics, GA, USA). X-ray photoelectron spectroscopy (XPS) was carried out on an Axis Ultra DLD system (Kratos, Manchester, UK) with a monochromatic Al Kα X-ray source. The samples were etched in 20 nm depth before XPS testing and the XPS data is calibrated using C 1s 285 eV. The loading mass of NiSe was measured using a thermogravmetric analyzer (TGA)/differential scanning calorimetry (DSC) analyzer (Netzsch Instruments, STA 449 F5, Selb, Germany) at a heating of 10 °C min^{-1} in air from room temperature to 800 °C, which is further explained in the discussion section.

Electrochemical Measurements

The working electrode was fabricated by dispersing the as-obtained materials with super P and sodium carboxymethyl cellulose (CMC) in a mass ratio of 80:10:10. The resultant slurry was pasted onto copper foil, dried at 100 °C in a vacuum oven for 12 h, which was followed by being pressed at 200 kg cm^{-2} and then cut into disk-shape electrode films (diameter, ~11 mm). The loading mass of each electrode film was 1.5~1.7 mg cm^{-2}. Electrochemical measurements were characterized by using two-electrode coin cells (CR2016) which were assembled in an argon filled glovebox. Sodium/Lithium metal and Celgard 2400 membrane were used as counter electrode and separator, respectively. The electrolyte solution was composed of 1 M $NaPF_6$/$LiPF_6$ dissolved in a mixture of ethylene carbonate (EC) and diethylcarbonate (DEC) (EC:DEC, 1:1 in volume) with the addition of 10 wt% fluoroethylene carbonate (FEC). Cyclic voltammogram (CV) measurements were performed on a CHI 660E electrochemistry workstation with a scan rate of 0.1 mV s^{-1} within the potential range of 0 to 3 V versus Na/Li metal at room temperature. The galvanostatic charge-discharge measurements were carried out at room temperature with cutoff voltages of 0~3.0 V using a LAND battery test system (Wuhan, China). Electrochemical impedance spectra (EIS) were performed on a CHI 660E electrochemical workstation with the frequency from 100 KHZ to 0.1 HZ.

2. XRD Pattern of rGO.

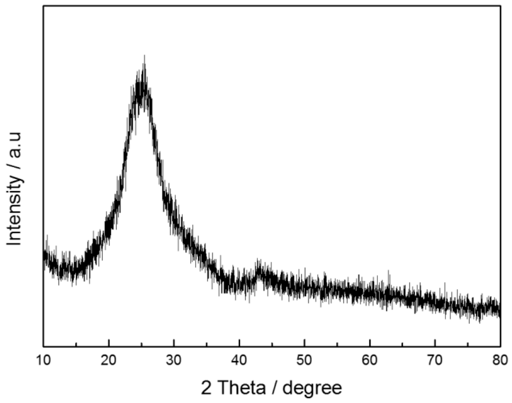

Figure S1. XRD pattern of rGO.

As shown in Figure S1, there is a big hump in 20°~30° which is consistent with the XRD pattern of NiSe/rGO.

3. High-Resolution XPS Spectrum of Se 3d and C 1s for NiSe/rGO.

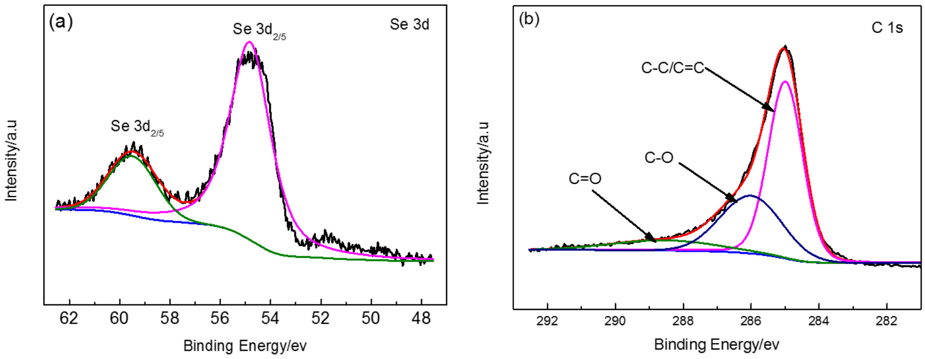

Figure S2. High-resolution XPS spectrum of (**a**) Se 3d and (**b**) C 1s for NiSe/rGO.

4. Raman Spectra of GO and NiSe/rGO

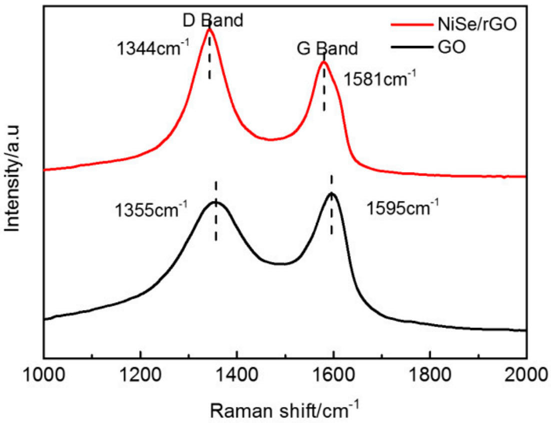

Figure S3. Raman spectra of GO and NiSe/rGO.

5. N_2 Adsorption–Desorption Isotherm of Bare NiSe and NiSe/rGO

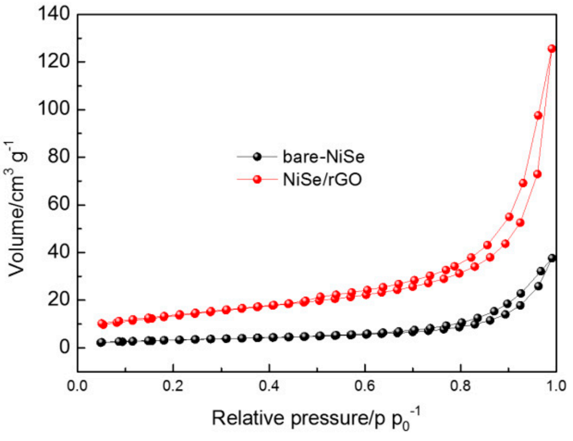

Figure S4. N_2 adsorption–desorption isotherm of bare NiSe and NiSe/rGO.

6. TEM Image of Bare NiSe and High Magnification SEM Image of NiSe/rGO

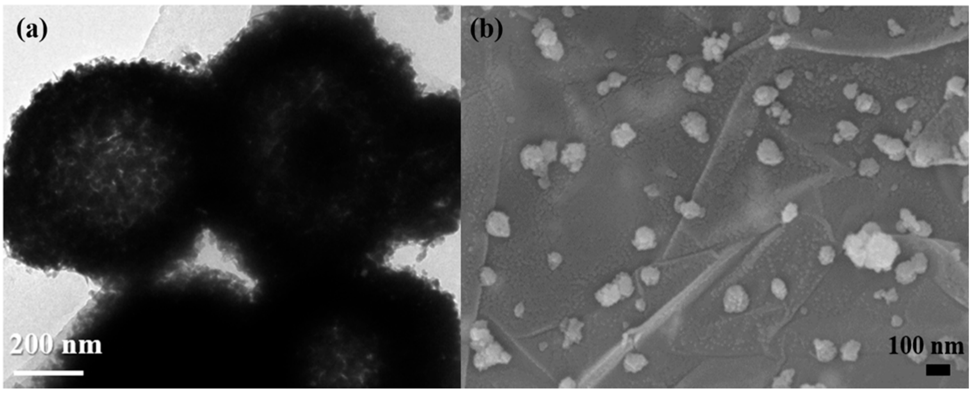

Figure S5. (**a**) TEM image of bare NiSe and (**b**) HRSEM image of NiSe/rGO.

7. The Selected Area Electron Diffraction (SAED) Pattern of NiSe/rGO and The Related Structrual Parameters

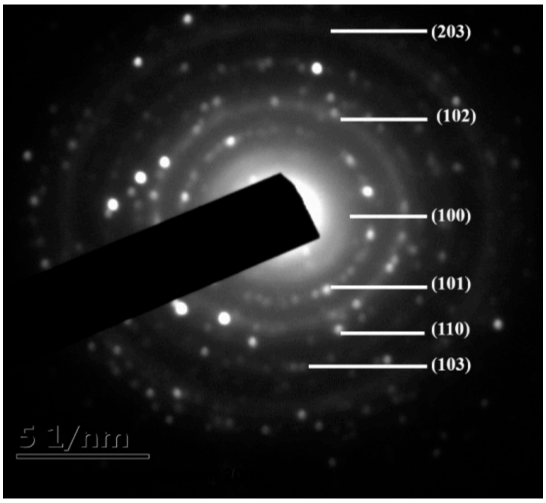

Figure S6. Selected area electron diffraction (SAED) pattern of NiSe/rGO.

Table S1. Comparison of the measured d-spacing and angles with the ideal structure.

Lattice Plane	Measure		Ideal	
	d-Spacing/nm	Angles/Degree	d-Spacing/nm	Angles/Degree
(100)	0.334	13.36	0.32	13.93
(101)	0.281	15.9	0.273	16.39
(102)	0.212	21.3	0.204	22.18
(110)	0.19	23.91	0.183	24.97
(103)	0.163	28.2	0.155	29.78
(203)	0.124	38.34	0.118	40.71

The measured d-spacing and diffraction angles are obtained by measuring the radius of the polycrystalline rings and then calculating from Bragg equation.

8. CV and Galvanostatic Discharge/Charge Voltage Profiles of Bare NiSe for NIBs.

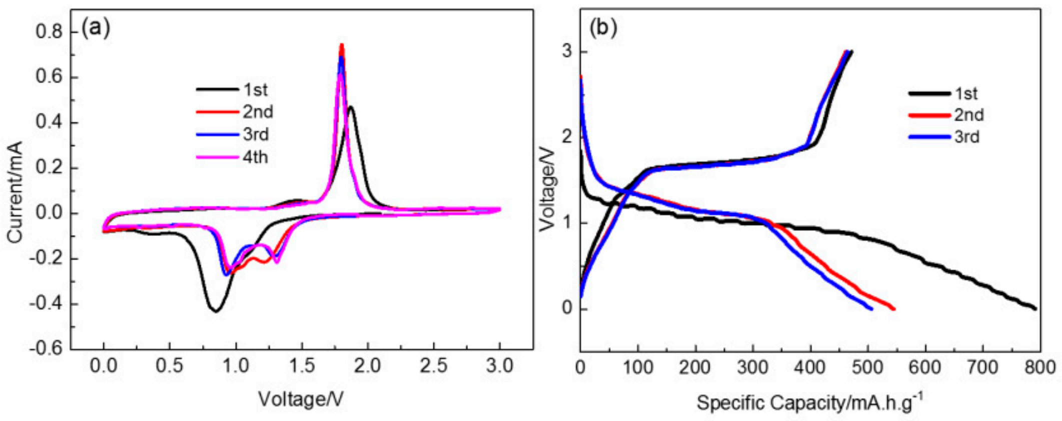

Figure S7. (**a**) CV curves obtained at a scan rate of 0.1 mV s^{-1} and (**b**) charge-discharge profiles at a current density of 0.05 A g^{-1} of bare NiSe electrode for SIBs.

9. CV and Galvanostatic Discharge/Charge Voltage Profiles of Bare NiSe for LIBs

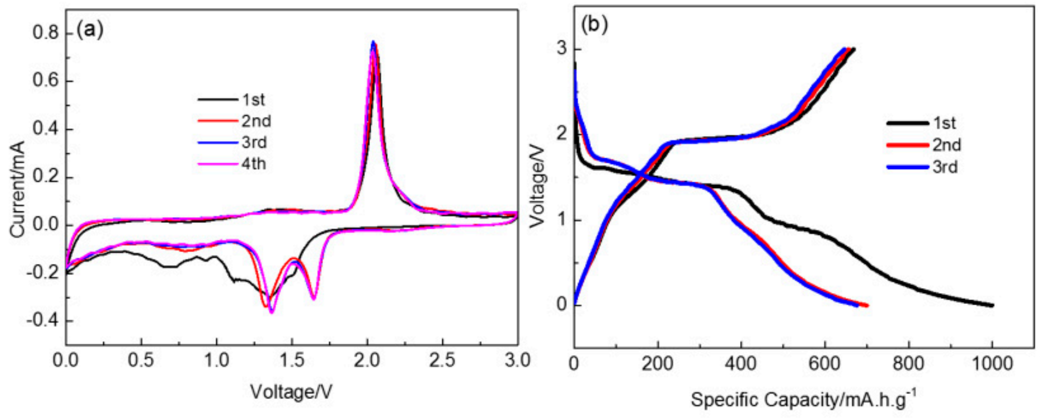

Figure S8. (**a**) CV curves obtained at a scan rate of 0.1 mV s^{-1} and (**b**) charge-discharge profiles at a current rate of 0.05 A g^{-1} of bare NiSe electrode for LIBs.

10. The XRD Pattern of NiSe/rGO Electrode of SIB at Fully-Charged-State after 50 Cycles.

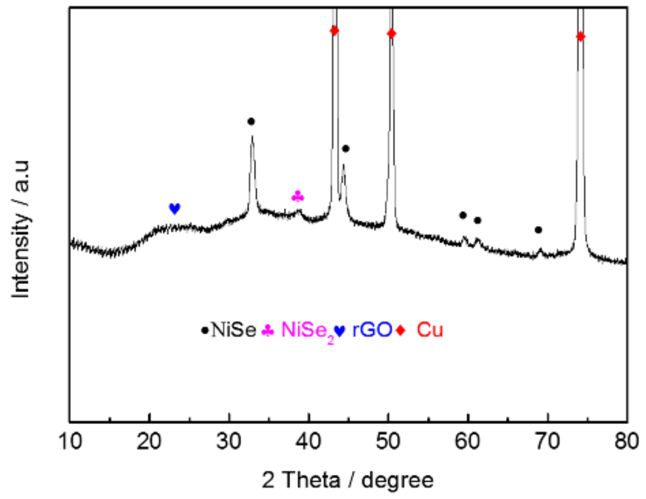

Figure S9. The XRD pattern of NiSe/rGO electrodes at fully-charged-state after 50 cycles in SIBs.

11. The SEM Images of Bare NiSe and NiSe/rGO Electrodes before and after 50 Cycles in SIBs.

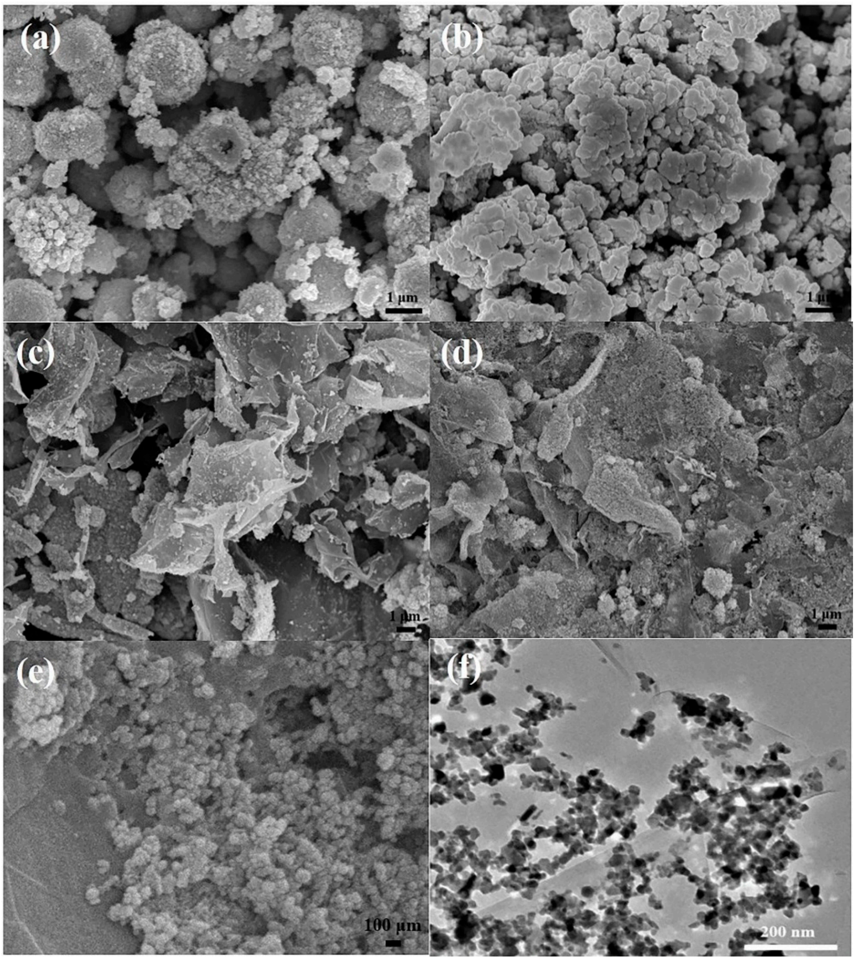

Figure S10. SEM images of bare NiSe and NiSe/rGO electrodes before (**a**,**c**) and after 50 cycles (**b**,**d**–**e**), TEM image of NiSe/rGO electrodes after 50 cycles (**f**) in SIBs.

12. Comparison of The Results in This Study with Those of other Materials in The Literature.

Table S2. Comparison of the results in this study with those of other materials in the literature.

Anode	Capacity/Current Density(LIBs)	Capacity/Current Density(SIBs)	Reference
NiSe/rGO	1125 mA h g^{-1}/0.05 A g^{-1}	423 mA h g^{-1}/0.05 A g^{-1}	This work
carbon nanobubbles	1394 mA h g^{-1}/1 A g^{-1}	175 mA h g^{-1}/0.05 A g^{-1}	[1]
Co_3O_4 microspheres	1148 mA h g^{-1}/0.2C(1C = 890 mA g^{-1})	290 mA h g^{-1}/0.2C(1C = 890 mA g^{-1})	[2]
hollow carbon spheres	512 mA h g^{-1}/1.2C(1C = 372 mA g^{-1})	120 mA h g^{-1}/0.2 A g^{-1}	[3]
NiS_2@CoS_2 nanocrystals	600 mA h g^{-1}/1 A g^{-1}	600 mA h g^{-1}/1 A g^{-1}	[4]
Ge@Graphene@TiO_2 Nanofibers	1050 mA h g^{-1}/0.1 A g^{-1}	182 mA h g^{-1}/0.1 A g^{-1}	[5]
Mo_3Sb_7	430 mA h g^{-1}/3719 mA h cm^{-3}	330 mA h g^{-1}/2854 mA h cm^{-3}	[6]
a-Fe_2O_3 microspheres	1440.9 mA h g^{-1}/0.4 A g^{-1}	129 mA h g^{-1}/0.1 A g^{-1}	[7]
N-doped TiO_2 nanorods	262 mA h g^{-1}/2C(1C = 168 mA g^{-1})	166 mA h g^{-1}/5C(1C = 168 mA g^{-1})	[8]
$MoSe_2$ nanosheets	684 mA h g^{-1}/1 A g^{-1}	580 mA h g^{-1}/0.2 A g^{-1}	[9]

References

1. Song, H.; Li, N.; Cui, H.; Wang, C. Porous Enhanced storage capability and kinetic processes by pores- and hetero-atoms- riched carbon nanobubbles for lithium-ion and sodium-ion batteries anodes. *Nano Energy* **2014**, *4*, 81–87.
2. Wen, J.; Zhang, D.; Zang, Y.; Sun, X.; Cheng, B. Li and Na storage behavior of bowl-like hollow Co_3O_4 microspheres as an anode material for lithium-ion and sodium-ion batteries. *Electrochim. Acta* **2014**, *132*, 193–199.
3. Zhang, K.; Li, X.; Liang, J.; Qian, Y. Nitrogen-doped porous interconnected double-shelled hollow carbon spheres with high capacity for lithium ion batteries and sodium ion batteries. *Electrochim. Acta* **2015**, *155*, 174–182.
4. Lin, Y.; Qiu, Z.; Li, D.; Zhu, C. NiS_2@CoS_2 nanocrystals encapsulated in N-doped carbon nanocubes for high performance lithium/sodium ion batteries. *Energy Storage Mater.* **2018**, *11*, 67–74.
5. Wang, X.; Fan, L.; Gong, D.; Lu, B. Core–Shell Ge@Graphene@TiO_2 nanofibers as a high capacity and cycle-stable anode for lithium and sodium ion battery. *Adv. Funct. Mater.* **2016**, *26*, 1104–1111.
6. Baggetto, L.; Allcorn, E.; Manthiram, A. Mo_3Sb_7 as a very fast anode material for lithium-ion and sodium-ion batteries. *J. Mater. Chem. A* **2013**, *1*, 11163–11169.
7. Wu, Z.; Zhong, Y.; Liu, J.; Yang, Z. Subunits controlled synthesis of a-Fe_2O_3 multishelled core–shell microspheres and their effects on lithium/sodium ion battery performances. *J. Mater. Chem. A* **2015**, *3*, 10092–10099.
8. Yang, Y.; Ji, X.; Jing, M.; Banks, C.E. Carbon dots supported upon N-doped TiO_2 nanorods applied into sodium and lithium ion batteries. *J. Mater. Chem. A* **2015**, *3*, 5648–5655.
9. Yang, X.; Zhang, Z.; Fu, Y.; Li, Q. Porous hollow carbon spheres decorated with molybdenum diselenide nanosheets as anodes for highly reversible lithium and sodium storage. *Nanoscale* **2015**, *7*, 10198–10203.

© 2019 by the authors. Submitted for possible open access publication under the terms and conditions of the Creative Commons Attribution (CC BY) license (http://creativecommons.org/licenses/by/4.0/).
